# Radiation-Induced Alterations in Cancer-Associated Fibroblasts: Drivers of Tumor Radioresistance and Therapeutic Targets

**DOI:** 10.3390/biom16060777

**Published:** 2026-05-26

**Authors:** Zheng Shi, Cuilan Hu, Chao Sun

**Affiliations:** 1 College of Biopharmaceutical and Engineering, Lanzhou Jiaotong University, Lanzhou 730000, China; 2 Institute of Modern Physics, Chinese Academy of Sciences, Lanzhou 730000, China; hucuilan23@mails.ucas.ac.cn (C.H.); sunchao@impcas.ac.cn (C.S.); 3 Key Laboratory of Heavy Ion Radiation Biology and Medicine of Chinese Academy of Sciences, Lanzhou 730000, China

**Keywords:** cancer-associated fibroblasts, radiotherapy, radioresistance, tumor microenvironment

## Abstract

Radiotherapy serves as a cornerstone of cancer treatment, but its efficacy is often compromised by radioresistance, a process in which cancer-associated fibroblasts (CAFs) play a critical role. Following irradiation, CAFs exhibit inherent radioresistance, not only surviving at higher doses but also undergoing profound functional reprogramming, including senescence, acquisition of a senescence-associated secretory phenotype (SASP), and myofibroblast activation. Importantly, CAFs employ multiple interconnected mechanisms to collectively drive radioresistance: sustained immunosuppression, pro-resistance paracrine signaling, exosome-mediated communication, and stromal remodeling. These reprogrammed CAFs create a microenvironment that paradoxically supports tumor recurrence and limits therapeutic efficacy. Intervention strategies targeting CAFs—including neutralizing soluble factors, blocking key signaling nodes, targeted therapies against fibroblast activation proteins, or disrupting exosome-mediated communication—have shown promise in preclinical studies. A deeper understanding of the complex interactions between radiotherapy and CAFs may ultimately drive a shift in therapeutic strategy from targeting tumor cells alone to leveraging the entire microenvironment to achieve durable antitumor effects.

## 1. Introduction

Radiotherapy is a cornerstone of comprehensive cancer treatment; more than 50% of cancer patients receive radiation therapy at various stages of their disease [1]. Radiotherapy primarily kills tumor cells by directly damaging their DNA through ionizing radiation, or indirectly causing DNA damage through free radicals generated by water ionization [2]. Based on the “4R” principles of radiation biology—repair, repopulation, redistribution, and reoxygenation—clinical radiotherapy protocols have been continuously optimized, resulting in effective tumor control [3].

However, the biological effects of radiotherapy extend far beyond the direct killing of tumor cells. As a physical stressor, ionizing radiation simultaneously acts on all components of the tumor microenvironment (TME), including blood vessels, immune cells, and fibroblasts [4,5]. The TME is central to tumor progression, immune evasion, and treatment response [6,7]. In recent years, with a deeper understanding of “side effects” and “distant effects,” research has revealed that radiotherapy can both induce immunogenic cell death and activate antitumor immunity, while also potentially triggering chronic inflammation and immunosuppression, which may conversely promote tumor regrowth [8,9]. This dual effect suggests that a deeper understanding of the remodeling of the TME by radiotherapy is crucial for optimizing radiotherapy strategies and overcoming radioresistance.

Among the numerous components of the TME, cancer-associated fibroblasts (CAFs) play a crucial role as a highly functionally heterogeneous core component [10]. CAFs are one of the most heterogeneous stromal cell populations in the TME [11]. Due to the lack of a specific single marker, the identification of CAFs typically relies on a combination of multiple markers, including α-smooth muscle actin (α-SMA), fibroblast activation protein (FAP), platelet-derived growth factor receptor α/β (PDGFRα/β), and fibroblast-specific protein 1 (FSP1) [12,13]. It is worth noting that none of these markers possesses absolute specificity, and their expression patterns vary across different tumor types and CAF subpopulations.

The origin of CAFs is highly heterogeneous, and this diversity in origin underlies their functional heterogeneity [14]. Resting normal fibroblasts or astrocytes are the primary sources of CAFs; they are activated and transdifferentiated into CAFs upon stimulation by tumor-secreted cytokines, such as Transforming growth factor-β (TGF-β) and Leukemia Inhibitory Factor (LIF) [15,16]. In addition, bone marrow-derived mesenchymal stem cells (BM-MSCs) can be recruited to the tumor site and differentiate into CAFs [17]. Epithelial cells can acquire a fibroblast phenotype through epithelial–mesenchymal transition (EMT) [18], and endothelial cells can do so through endothelial-mesenchymal transition (EndMT) [19]. Less common cellular precursors, including adipocytes, pericytes, and mesothelial cells, also possess the potential to differentiate into CAFs [20,21]. This multi-lineage origin results in a rich phenotypic spectrum of CAFs within the TME.

At the phenotypic level, CAFs exhibit high diversity. Myofibroblast-like CAFs (myCAFs) are one of the major subtypes of CAFs and are particularly prominent in pancreatic ductal adenocarcinoma (PDAC) and head and neck squamous cell carcinoma (HNSCC); they are characterized by moderate expression of integrin α11β1 [22]. Inflammatory CAFs (iCAFs) are another major subtype; in non-small cell lung cancer (NSCLC), they promote brain metastasis by activating the Mesenchymal–epithelial transition Factor (MET)/Hepatocyte growth factor (HGF) signaling pathway [23], and their presence is associated with an inflammatory TME and higher patient survival rates [24]. In addition, antigen-presenting CAFs (apCAFs) have been identified as a new subpopulation; their markers, UBE2T and KPNA2, promote CAF invasiveness [25] and may participate in immune regulation through CD8^+^ T cell captures and regulatory T cell differentiation [26]. Matrix CAFs (mCAFs), on the other hand, are associated with low immune infiltration and poor prognosis [24]. Beyond these well-characterized subtypes, additional CAF subpopulations with distinct roles in the context of radiotherapy have been identified. A specific CAF subset regulated by interferon signaling—ilCAFs (characterized by high IRF1 expression)—is positively correlated with radiosensitivity in multiple solid tumors [27]. Conversely, a radioresistant CAF subset—CAF^R^—enriched in colorectal cancer promotes resistance by secreting high levels of TGF-β1, inducing WARS2-IT1 expression, stabilizing HIF-1α, and activating glycolysis [28].

From a functional perspective, the functional diversity of CAFs makes them a key regulatory hub within the TME. On the one hand, CAFs directly promote tumor progression through multiple mechanisms [29,30]. Regarding extracellular matrix remodeling, CAFs are major producers of matrix collagen; through secreting matrix metalloproteinases and lysyl oxidases, they alter matrix stiffness, tension, and tissue structure, forming physical barriers and creating pathways conducive to tumor invasion [31]. In terms of angiogenesis regulation, CAFs secrete pro-angiogenic factors such as Vascular endothelial growth factor (VEGF) and Fibroblast growth factor (FGF), promoting tumor angiogenesis [32]. Regarding metabolic support, CAFs can sustain tumor cell energy metabolism by providing metabolic substrates like lactate and glutamate [33]. On the other hand, CAFs serve as the central engine in shaping the immunosuppressive microenvironment. Through secretion of various cytokines and chemokines, they recruit and reprogram immune cells, suppress effector T-cell activity, and assist the tumor in achieving immune evasion [33,34,35,36].

In summary, CAFs play a complex and multifaceted role in tumor initiation and progression, and their functions are highly environment-dependent. The functional state and dynamic evolution of CAFs profoundly influence radiation sensitivity. Under the influence of radiotherapy, a potent stressor, CAFs serve both as recipients of radiation-induced damage and as key drivers of TME remodeling. A deeper understanding of the regulatory mechanisms by which radiotherapy modulates CAFs is of great significance for elucidating radioresistance and developing new therapeutic strategies. This review systematically elucidates the regulatory effects of radiotherapy on CAFs, covering the direct biological effects of radiation on CAFs, the functional reprogramming of CAFs following radiotherapy, the heterogeneity of CAFs and their differential responses to radiotherapy, as well as strategies for sensitizing radiotherapy by targeting CAFs, with the aim of providing a theoretical basis for optimizing the efficacy of radiotherapy.

## 2. Radiation-Induced Alterations in CAFs: From Survival to Activation

**Figure 1:**
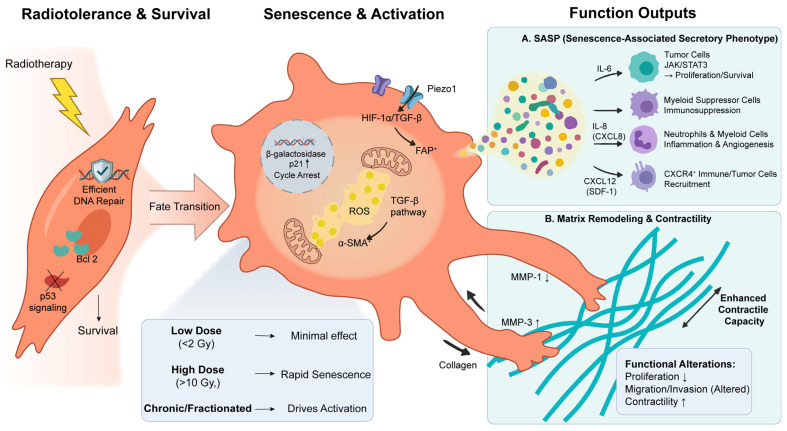
The Effects of Radiotherapy on CAFs. CAFs survive after radiotherapy due to their unique radioresistance, but they do not remain unchanged—they enter a senescent state, acquire the SASP, and simultaneously exhibit an activated phenotype. Specifically: radiotherapy induces senescence in CAFs, manifested by cell cycle arrest; Senescent CAFs secrete factors such as IL-6 and IL-8 via the SASP, activating the JAK/STAT3 pathway to promote tumor cell proliferation and survival, and recruit immunosuppressive cells; in terms of activation, radiotherapy drives the upregulation of α-SMA through ROS/TGF-β and Piezo1/HIF-1α pathways. Functionally, CAFs exhibit enhanced contractile capacity, reduced proliferative capacity, and altered migration/invasion capabilities.

The primary effect of radiotherapy on CAFs is to determine their cellular fate. CAFs survive after radiotherapy due to their unique radiation resistance, but they do not remain in their original state: they enter a senescent state, acquire a senescence-associated secretory phenotype (SASP), and simultaneously exhibit an activated phenotype, with profound changes in their proliferation, migration, and contractile functions. This section will discuss the direct regulatory effects of radiotherapy on CAFs from multiple perspectives, as shown in Figure 1.

### 2.1. Radiotolerance and Senescence

CAFs exhibit unique radioresistance, surviving at clinical radiation doses [37]. Multiple studies have confirmed that human CAFs remain viable and persist in vitro even after irradiation with an ablative dose of 18 Gy [38]; some studies have even observed that CAFs can tolerate radiation doses as high as 30 Gy without undergoing apoptosis [5]. The mechanism underlying this radioresistance remains poorly understood but may involve multiple aspects: CAFs may possess more efficient DNA repair capabilities [39]; they may be more resistant to checkpoint protein-mediated cell cycle arrest [39]; or may be less prone to apoptosis due to impaired p53 signaling or high expression of pro-survival proteins from the Bcl-2 family [40]. This unique radioresistance implies that after radiotherapy, when a large number of tumor cells are eliminated, CAFs may persist as “survivors” within the TME and continue performing their functions.

Although CAFs are able to evade radiation-induced apoptosis, they do not emerge entirely unscathed. Studies have shown that when radiation doses exceed a specific threshold of 10–12 Gy, CAFs enter a state of senescence [5]. At a dose of 18 Gy, CAFs rapidly senesce within days of irradiation; although they maintain the DNA damage response, they can still survive long-term in tissue culture. This phenomenon is referred to as stress-induced premature senescence [38]. Aging CAFs exhibit characteristic morphological changes—cell flattening and enlargement, increased β-galactosidase activity, and permanent cell cycle arrest [41,42]. CAFs derived from colorectal and lung cancers both exhibited reduced proliferative capacity and upregulation of the cell cycle arrest protein p21 following irradiation, suggesting that they entered a state of growth arrest without undergoing cell death or morphological changes [43,44]. Studies on colorectal cancer have also found that radiotherapy induces interleukin (IL)-1α to drive CAFs toward an inflammatory phenotype (iCAFs) and triggers p53-mediated senescence, enabling iCAFs to acquire functions that promote radioresistance [45]. Studies on NSCLC indicate that these senescent-like CAFs actually promote tumor cell proliferation and resistance to radiotherapy via the janus kinase/signal transducer and activator of transcription (JAK/STAT) pathway, which is a major cause of radiotherapy failure [46].

### 2.2. Senescence-Associated Secretory Phenotype

It is worth noting that cellular senescence is not merely a state of functional quiescence. Senescent cells remain metabolically active and communicate with other cells by secreting growth factors and inflammatory cytokines; this phenomenon is known as the SASP and is associated with various disease processes [47,48], and serves as a key biological basis for distant effects following radiotherapy, tumor recurrence, and treatment resistance [43]. The changes in the secretome of radiation-induced CAFs are diverse and complex. Cytokines and chemokines are the most extensively studied class of molecules within SASP. IL-6 is a core component of the SASP, and radiation-exposed CAFs persistently secrete IL-6 [43]. IL-6 directly promotes tumor cell proliferation and survival by activating the JAK/STAT3 pathway, while also recruiting myeloid suppressor cells to shape an immunosuppressive microenvironment [49]. IL-8, as known as C-X-C motif chemokine ligand (CXCL) 8, is also upregulated in irradiated CAFs. As a potent chemokine for neutrophils and myeloid cells, IL-8 recruits these cells into the TME, promoting inflammatory responses and angiogenesis [50,51]. CXCL12, known as Stromal Cell-Derived Factor-1 (SDF-1), is one of the signature secreted factors of CAFs and is involved in the recruitment of C-X-C chemokine receptor type 4 (CXCR4)-expressing immune cells and tumor cells [52].

### 2.3. Phenotypic and Functional Alterations

Radiation not only affects the survival of CAFs but also regulates their activated phenotype. Irradiated fibroblasts exhibit typical myofibroblastic characteristics: upregulated α-SMA expression, increased stress fiber formation, and enhanced contractile capacity [53]. The acquisition of this activated phenotype involves the Transforming Growth Factor-β (TGF-β) signaling pathway. Radiotherapy activates the TGFβ signaling pathway by inducing the accumulation of reactive oxygen species (ROS) in mitochondria, thereby promoting the transformation of normal fibroblasts into α-SMA^+^ muscular fibroblast/CAF phenotypes [54]. Furthermore, post-radiotherapy extracellular matrix remodeling activates the membrane mechanosensor Piezo1 on CAFs, driving the activation of FAP^+^ CAFs via the hypoxia-inducible factor-1α (HIF-1α)/TGF-β pathway, thereby exacerbating the fibrotic process [53]. Notably, external physical stimuli such as blue light irradiation have been shown to suppress the activated state of CAFs, suggesting that physical factors have the potential to bidirectionally regulate the CAF phenotype [55]. Evidence regarding the reversibility of radiation-induced CAF activation remains inconclusive. Some studies suggest that the activated state of CAFs may require sustained microenvironmental signaling for maintenance. For example, inhibition of the JAK/STAT signaling pathway can reverse LIF-induced persistent CAF activation [16]. However, radiation-induced senescence is a permanent cell cycle arrest; once cells enter the senescent state, they are unlikely to fully revert to the quiescent phenotype [56,57]. This implies that radiotherapy may lead to long-term or even permanent alterations in the CAF phenotype within the TME.

Radiation has a significant inhibitory effect on the proliferative capacity of CAFs. Unlike tumor cells, CAFs enter a state of cell cycle arrest following irradiation, resulting in a substantial decline in their proliferative capacity [58], which is consistent with observations that they enter a senescent state. Changes in their migratory and invasive capabilities, however, are more complex. Hellevik et al. reported that human CAFs exposed to 18 Gy of radiation exhibited reduced migration and invasion capabilities in vitro, which may be related to changes in the matrix metalloproteinase (MMP) expression profile: MMP-1 expression was downregulated in irradiated CAFs, while MMP-3 expression was upregulated. Furthermore, irradiated CAFs overexpress integrins α2β1 (collagen receptor) and α5β1 (fibronectin receptor), suggesting altered adhesion properties to the extracellular matrix [38]. Regarding contractile capacity, due to upregulated α-SMA expression and stress fiber formation, irradiated CAFs typically exhibit enhanced contractile function, which may affect the physical properties of the matrix [59].

### 2.4. Dose and Fractionation Effects

The dose–response relationship of ionizing radiation on CAFs exhibits complex, nonlinear characteristics. Ragunathan et al. proposed a framework: low-dose irradiation (<2 Gy) has relatively limited direct effects on CAFs; moderate doses (2–10 Gy) strike a balance between inducing immunogenic cell death and maintaining CAF function; high doses (>10 Gy), while capable of inducing tumor cell death, may also be accompanied by more extensive tissue damage and pro-inflammatory effects [5,60]. Radiation delivery patterns exert differential effects on fibroblast activation. Studies have shown that chronic radiation (2.5 Gy) can induce the transformation of healthy human fibroblasts into a CAF-like phenotype (upregulation of α-SMA), whereas acute radiation (<2.5 Gy) does not produce this effect [61]. Long-term fractionated radiotherapy can also drive the transformation of normal fibroblasts into the CAF phenotype by activating the TGF-β signaling pathway [54]. These radiation-induced CAFs accelerate tumor growth through mechanisms such as promoting angiogenesis [54].

In summary, the regulation of CAFs by radiotherapy is a complex, multidimensional, and dynamically evolving process. CAFs survive post-radiation therapy due to their unique radioresistance, but this “survival” is not static-they undergo a fate transition from senescence to activation, with their functions profoundly reshaped. Through the SASP, CAFs release a large number of inflammatory and immunomodulatory factors while exhibiting a myofibroblast-like activated phenotype, and their functions—including proliferation, migration, and contraction—are consequently altered. These changes mean that after radiotherapy, CAFs may either inhibit angiogenesis and tumor cell migration or promote tumor recurrence through immunosuppression and metabolic support; the ultimate outcome depends on a complex interplay of multiple factors, including radiation dose and fractionation, as well as the origin and subpopulation characteristics of the CAFs.

## 3. CAFs as Central Hubs of Radioresistance: Multifaceted Mechanisms in the TME

**Figure 2:**
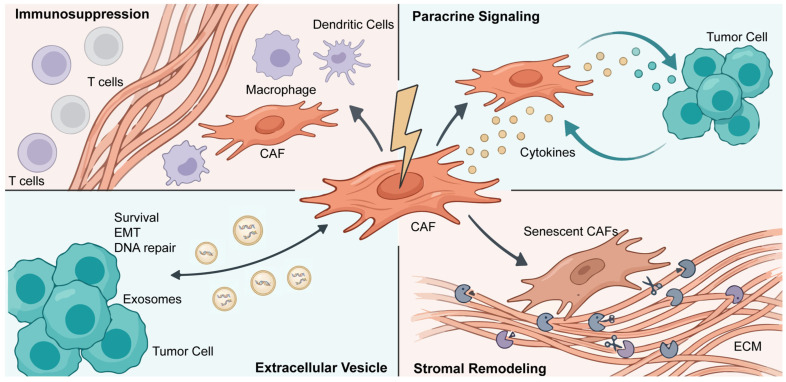
Mechanisms of CAF mediates radioresistance in the TME. Immunosuppression: CAFs suppress antitumor immune responses by influencing dendritic cells, T cells, and macrophages. Paracrine signaling: CAFs secrete cytokines to engage in bidirectional communication with tumor cells, thereby regulating tumor cell function. Extracellular vesicles: Through exosomes, CAFs promote tumor cell survival, EMT, and DNA repair. Matrix remodeling: Senescent CAFs alter the structure and function of the tumor microenvironment by remodeling the ECM.

Unlike normal fibroblasts, CAFs generally exert immunosuppressive effects in the TME, and their abundance is closely associated with poor treatment outcomes and negative prognosis [62,63,64]. Following radiotherapy, the function of CAFs is further reshaped, synergistically mediating treatment resistance through multiple mechanisms. From a functional perspective, these mechanisms can be categorized into four core axes, as shown in Figure 2: persistent immunosuppression, paracrine survival signals, extracellular vesicle communication, and matrix remodeling and side effects. The following discussion will focus on these four functional axes, attempting to distill common patterns from existing research while identifying key questions that remain to be answered.

### 3.1. Sustained Immunosuppression

It has been repeatedly demonstrated that CAFs inherently resist the “immune-activating effect” expected from radiotherapy. Even when exposed to high doses of radiation, CAFs continue to secrete inhibitory molecules such as prostaglandin E2 (PGE2) and IL-10, strongly suppressing T-cell activity, and do not release immunogenic death signals [65]. This phenomenon has been reported in various solid tumors, suggesting that it may be a relatively conserved mechanism [66,67].

CAF exerts multi-target regulation on immune cells, leading to convergent functional outcomes. Within T cells, radiation can induce upregulation of the co-inhibitory molecules CD73 and CD276 on the surface of CAFs, further promoting the formation of an immunosuppressive microenvironment [68]. At the macrophage level, CAFs exhibit bidirectional regulatory effects: on one hand, they induce undifferentiated macrophages to adopt a mixed M1/M2 phenotype; on the other hand, they significantly suppress the pro-inflammatory functions of M1 macrophages, including NO production, inflammatory cytokine secretion, and migratory capacity. Notably, this immunomodulatory capacity is tolerant to both single high-dose and fractionated radiotherapy [69]. Regarding dendritic cells, CAFs impair antigen presentation and T-cell activation by inducing a tolerant phenotype [70]. Furthermore, post-radiotherapy CAFs participate in immune evasion processes, further weakening the antitumor immune response [5]. In addition, CAFs can also interact with the extracellular matrix to form a dual physical and immunological barrier. Studies have shown that CAFs and collagen, through focal adhesion kinase (FAK)-mediated mechanisms, suppress radiation-induced interferon signaling and T-cell activation, thereby limiting the infiltration of immune cells into the tumor stroma [71].

Although the regulatory mechanisms of different immune cell subtypes have been described individually, it remains unclear how CAFs coordinate these multi-target effects simultaneously. Is there an upstream “master regulator” that uniformly drives these immunosuppressive programs? Or do CAFs act on different immune cells through multiple distinct pathways? Current research has not yet answered this question, and the answer may depend on the specific source and activation state of the CAFs.

### 3.2. Paracrine Signaling: CAF-Derived Factors Driving Radioresistance

Following radiotherapy, CAFs secrete a variety of soluble factors, establishing a complex paracrine dialogue with tumor cells that directly enhances the latter’s survival, proliferation, and invasive capacity. The heterogeneity of CAFs is particularly evident in the diversity of their secretory profiles: CAFs from different subpopulations or different tumor backgrounds influence the response to radiotherapy in their own unique ways.

From the perspective of functional output, paracrine signals derived from CAFs can be categorized into several groups. The first category is autophagy-promoting signals: CAFs secrete factors such as insulin-like growth factor (IGF)-1/2, CXCL12 and β-hydroxybutyrate, which promote autophagy in tumor cells and accelerate their recovery and regeneration following radiotherapy [72]. The second category is EMT-driving signals: chemokines such as CXCL12 and CXCL1 produced by CAFs can directly drive epithelial–mesenchymal transition in tumor cells, thereby conferring radioresistance [73,74]. In addition, more specific regulatory pathways have been reported in various types of cancer—for example, in hepatocellular carcinoma, CAFs enhance radioresistance by upregulating SOX4 [75]; in pancreatic cancer, TGFβ secreted by CAFs is also involved in this process [76].

Beyond these relatively isolated studies, a series of more specific signaling mechanisms further amplify the resistance-promoting effects of CAFs. In terms of regulating tumor cell survival and death, conditioned medium obtained from co-culturing cancer cells with CAFs can significantly enhance the radiation resistance of irradiated cervical cancer cells. This mechanism is associated with the synergistic secretion of multiple growth factors by CAFs (including PDGF, VEGF, IGF2, and EGF) and the suppression of radiation-induced gene expression [77]. Persistent CAFs following radiotherapy mediate radiation tolerance by upregulating Fibulin-5, activating the Src-STAT3 pathway, and inhibiting ferroptosis in cancer cells [78]. In nasopharyngeal carcinoma, CAFs infiltrating tumor tissue after radiotherapy secrete IL-8, activate the Nuclear Factor kappa-B (NF-κB) pathway, and enhance the DNA damage repair capacity of tumor cells, thereby promoting their survival following radiotherapy [79].

Of particular note is that CAFs and tumor cells can form a positive feedback amplification loop—which may be one of the core design principles underlying CAF-mediated radioresistance. In esophageal squamous cell carcinoma (ESCC), collagen type 1 (Col1) secreted by CAFs not only directly enhances the DNA repair capacity of tumor cells but also induces tumor cells to secrete CXCL1; CXCL1, in turn, further activates CAFs via paracrine signaling, forming a closed amplification loop that constitutes a key mechanism of radioresistance [80]. radiotherapy induces CAFs to secrete iNOS/NO, which activates endogenous iNOS/NO signaling in tumor cells via the NF-κB pathway, similarly forming a positive feedback loop that drives microenvironmental acidification and inflammatory responses, thereby mediating radioresistance [81]. In lung cancer, CAFs produce kynurenine through TDO2-mediated tryptophan metabolism. This metabolite directly activates the threonine protein kinase (AKT) pathway in tumor cells, promoting proliferation and metastasis, while simultaneously inhibiting dendritic cell maturation and T-cell immunity; this process is further upregulated by tumor-derived galectin-1, creating a malignant tumor cycle [70]. The elucidation of these positive feedback mechanisms provides a theoretical basis for disrupting the synergistic resistance between CAFs and tumor cells.

### 3.3. Extracellular Vesicle-Mediated Intercellular Communication

In addition to soluble factors, exosomes—another important vector for intercellular communication—play a significant role in CAF-mediated radioresistance. Exosomes can carry bioactive molecules such as nucleic acids and proteins, enabling efficient information transfer between cells.

A common mechanism observed across multiple studies is that CAF-derived exosomes deliver specific miRNAs to target the PI3K/Akt pathway, thereby enhancing DNA damage repair capacity in tumor cells and inhibiting apoptosis. In ESCC, radiotherapy can induce CAFs to deliver miR-193a-3p to tumor cells via exosomes, thereby regulating the PTEN/Akt pathway and promoting EMT and metastasis [82]. In colorectal cancer, CAF-derived exosomes deliver miR-590-3p, which specifically inhibits chloride channel accessory 4 (CLCA4) and activates the PI3K/Akt pathway, thereby enhancing DNA repair, suppressing apoptosis, and ultimately mediating radioresistance [83]. Furthermore, CAF-derived exosomes can induce a stem cell-like phenotype in colorectal cancer cells by activating the TGF-β signaling pathway, thereby further enhancing their resistance to radiotherapy [84].

However, exosome-mediated communication is not unidirectional. Radiotherapy can also induce breast cancer cells to secrete extracellular vesicles carrying CD44v3; these vesicles, in turn, activate fibroblasts to produce IL-6, thereby promoting tumor survival and tumor stem cell expansion, and mediating acquired radioresistance [85]. This finding reveals a bidirectional communication network formed via exosomes between tumor cells and CAFs. More importantly, extracellular vesicles derived from CAFs exhibit an intrinsic resistance to radiation therapy itself. Proteomic analysis reveals that CAF-EVs carry a variety of pro-tumor and immunomodulatory molecules, and neither single-dose high-dose nor fractionated radiotherapy alters their secretion levels or protein loading [86]. This implies that radiotherapy cannot effectively disrupt the CAF-mediated exosome signaling network—even after treatment, this communication channel continues to function within the TME.

### 3.4. Stromal Remodeling and Bystander Effects

Radiotherapy not only affects the secretory function of CAFs but also reshapes their paracrine profile, thereby altering the overall physicochemical properties of the TME. These changes can be summarized in three interrelated dimensions: reprogramming of the angiogenic profile, long-range diffusion of paracrine signals, and senescence-related matrix remodeling. More importantly, radiotherapy may also fundamentally alter the ability of CAFs to respond to microenvironmental signals—rather than simply “activating” or “inhibiting” them.

With regard to the regulation of angiogenesis, the effect of radiotherapy on the secretory profile of CAFs is not simply an enhancement or suppression, but rather a directional reprogramming. Taking CAFs in lung cancer as an example, a lethal dose of radiotherapy (18 Gy) induces a shift in the secretory profile of lung cancer CAFs: downregulating pro-angiogenic molecules such as stromal cell-derived factor-1 (SDF-1), angiopoietin, and thrombospondin-2 (TSP-2), and upregulating basic fibroblast growth factor (bFGF), while inflammatory factors such as HGF and IL-6 remain unaffected. Functionally, the conditioned medium derived from irradiated CAFs has no direct effect on the proliferation or migration of tumor cells themselves, but it partially inhibits the migration of endothelial cells. This finding suggests that radiation therapy may indirectly influence the TME by regulating CAF-mediated angiogenesis, rather than acting directly on tumor cells [87].

In terms of the spread of paracrine effects, the impact of radiotherapy on the TME is not limited to the irradiated region itself. Radiotherapy-activated fibroblasts can induce genomic instability in squamous cell carcinoma (SCC) cells in non-irradiated regions through TGF-β1-mediated paracrine effects, and upregulate proliferation- and invasion-related molecules such as c-Met and the mitogen-activated protein kinase (MAPK) pathway, thereby enhancing their malignant phenotype [88]. In other words, CAFs act as “signal relay stations” that propagate radiological effects from the irradiated to the non-irradiated regions, amplifying the global impact of radiotherapy on the TME.

In terms of matrix remodeling, radiation-induced senescent CAFs play a significant role. By secreting MMPs and remodeling the cytoskeleton, these senescent cells disrupt the structural integrity and signaling homeostasis of the breast stromal microenvironment, thereby promoting the malignant transformation of epithelial cells and enhancing the invasive capacity of breast cancer cells [89].

Beyond the mechanisms described above, a more fundamental question arises: How exactly does radiotherapy alter the functional state of CAFs? Current evidence suggests that this alteration may be more complex than previously anticipated. Radiotherapy itself does not directly alter the expression or activation levels of PDGF/TGF-β receptors in CAFs; however, in the presence of ligands, irradiated CAFs exhibit impaired TGF-β downstream signaling (pSmad) [90]. This suggests that the remodeling of CAF function by radiation therapy is not simply a matter of “activation” or “inhibition,” but rather a reconfiguration of their ability to respond to microenvironmental signals. This finding offers a new perspective on the complexity of changes in CAF function following radiation therapy.

## 4. Therapeutic Strategies: Targeting CAFs to Overcome Radioresistance

Given the central role of CAFs in mediating radioresistance, targeting CAFs and their associated pathways has become a key strategy for radiosensitization. In recent years, researchers have developed various intervention methods, including soluble factors, signaling pathways, targeted delivery of FAPs, and exosome-mediated communication. For example, the CAF inhibitor Tranilast effectively blocks the protective effects of CAFs on tumor cells, thereby enhancing the efficacy of radiotherapy [79]. However, given the high heterogeneity of CAFs, a fundamental question persists: do these strategies actually eliminate CAFs, or do they merely reprogram their functions? Can the radiosensitizing effects observed in preclinical studies be translated into genuine clinical benefits? The following section categorizes existing strategies into four groups, presenting the evidence while focusing on the respective barriers to clinical translation.

### 4.1. Targeting CAF-Derived Soluble Factors

The TGF-β signaling pathway is one of the most widely studied targets. Bintrafusp alpha (BA), a bifunctional fusion protein, is a commonly used approach targeting TGF-β; it combines the TGF-β receptor II with an IgG1 monoclonal antibody directed against PD-L1, can synergistically remodel the immune microenvironment in the context of radiotherapy, increase T-cell infiltration, reduce radiotherapy-induced fibrosis, and eliminate treatment-resistant tumors while protecting normal tissues [91]. Whether used alone or in combination with other therapeutic strategies, this approach has demonstrated promising efficacy in preclinical studies and multiple clinical trials across various tumor types [76,92,93,94,95].

The IL-1 signaling pathway also plays a critical role in CAF-mediated radioresistance. Anti-IL-1β monoclonal antibodies enhance the efficacy of chemotherapy and anti-PD-1 therapy by inducing phenotypic shifts in the CAF population and reshaping the immune microenvironment [96]. Targeting IL-1 signaling or eliminating senescent iCAFs can reverse treatment resistance in colorectal cancer and enhance radiosensitivity [45]. Furthermore, the IL-1 inhibitor anakinra exhibits good synergistic effects with radiotherapy in vivo; based on these findings, a Phase I clinical trial targeting patients with rectal cancer has been initiated (NCT04942626) (NCT04942626) [97].

Chemokines and their receptors also show potential for therapeutic intervention. Inhibiting the CXCL12/CXCR4 interaction can attenuate the tumor-promoting function of CAFs induced by radiotherapy. In ESCC, the use of anti-CXCL1 antibodies can reverse the radioresistance mediated by CXCL1 secreted by CAFs [73]. IL-6 secreted by CAFs can induce radioresistance by activating the STAT3 signaling pathway in breast cancer cells, while the anti-IL-6 monoclonal antibody tocilizumab can block this effect [98].

In addition, metabolic factors also warrant attention. IGF1 secreted by radiation-induced CAFs drives metabolic reprogramming and metastasis by activating the IGF1R-mTOR pathway in tumor cells; targeting this axis can enhance the efficacy of radiotherapy for rectal cancer [44]. Radiotherapy can also activate CAFs to upregulate iNOS/NO secretion, driving the formation of an acidic microenvironment; combining iNOS inhibition with radiotherapy can sensitize pancreatic cancer [81].

A common challenge facing the above strategies is the significant functional overlap and compensatory mechanisms among CAF-derived soluble factors—if a single factor is blocked, will other factors rapidly upregulate to compensate? Systematic studies to address this question are currently lacking.

### 4.2. Targeting Key Mediators of CAF-Cancer Cell Crosstalk

Compared to targeting soluble factors, the mediator strategy targeting key nodes in signaling networks is theoretically more efficient. Secreted Frizzled-Related Protein 2 (SFRP2) and Plasminogen Activator Inhibitor-1 (PAI-1) play critical roles in the distant effects of radioimmunotherapy. Studies have shown that PAI-1 induces the differentiation of pericyte cells within distant tumors into SFRP2-high-expressing CAFs, thereby preventing CD8^+^ T cells from entering non-irradiated tumors. Pharmacological blockade of PAI-1 or SFRP2 can effectively reverse this process and enhance the distant effect [99].

Senescent-like CAFs are another key mediator. Radiation-induced senescent-like CAFs promote NSCLC cell proliferation and radioresistance via the JAK/STAT pathway. Targeting and eliminating senescent-like CAFs, such as using the FOXO4-p53 interfering peptide FOXO4-DRI, can simultaneously enhance radiosensitivity and reduce radiation-induced pulmonary fibrosis [46].

The integrin/FAK signaling axis plays a vital role in cell–matrix signaling. In pancreatic cancer, CAFs form an immune barrier through FAK-mediated synergistic interactions with collagen; FAK inhibition increases mouse sensitivity to radiotherapy by reprogramming CAFs [71]. Furthermore, targeting fibronectin-binding integrins with α5β1/αvβ3 bispecific antibodies can alter CAF-derived extracellular matrix assembly and suppress their pro-tumor effects [100].

In addition, precision subtyping based on CAF molecular characteristics also offers new insights for personalized radiotherapy. Based on transcriptomic changes in radiotherapy-induced CAFs, the researchers identified and validated CAF-associated genetic signatures capable of predicting the risk of biochemical recurrence and metastasis following radiotherapy for prostate cancer. The predictive model, constructed in combination with clinical factors, can accurately assess 2-, 3-, and 5-year survival rates, providing a new molecular tool for individualized risk assessment in radiotherapy patients [101]. This study suggests that the molecular characteristics of CAFs not only hold value for therapeutic targeting but can also serve as an important basis for patient stratification, which may be key to advancing precision interventions.

### 4.3. FAP-Targeted Therapies and Nanodelivery Systems

FAP is highly expressed on the surface of CAFs in various solid tumors but is expressed only to a limited extent in normal tissues, making it an ideal target for precisely targeting CAFs. In recent years, therapeutic strategies targeting FAP have become increasingly diverse.

Regarding FAP-targeted immunotherapy, studies have shown that radiotherapy can induce locally elevated FAP expression in tumors. Combining the FAP-targeted 4-1BBL fusion protein with local radiotherapy can achieve control of the primary tumor and distant effects by enhancing CD8^+^ T-cell activation and through Type I interferon and Interferon-γ (IFN-γ) signaling [102]. The combination of FAP-CD40 agonists and local radiotherapy also demonstrated significant synergistic efficacy in an HPV-positive HNSCC model; this effect depends on radiotherapy-induced TME remodeling and activation of the CD8^+^ T cell–conventional dendritic cell type 1 (cDC1) axis [103]. These studies precisely deliver immune agonists to CAF-rich regions, thereby enhancing therapeutic efficacy while avoiding the toxic risks associated with systemic immune activation.

The FAPα-targeted vaccine offers a new approach to activating antitumor immunity. The combination of stereotactic ablation radiotherapy (SABR) and the FAPα-targeted cancer vaccine demonstrated significant synergistic efficacy in a metastatic breast cancer model with low immunogenicity. This combination therapy not only inhibits primary tumor growth but also generates a distant anti-lung metastasis effect by activating CD8^+^ T cells, reprogramming myeloid-derived suppressor cells (MDSCs) and regulatory T cells (Tregs), and inducing M1 polarization of macrophages, thereby providing a new strategy for overcoming treatment resistance by targeting FAP^+^ CAFs [104].

Bionic nano delivery systems provide precise and efficient tools for targeting CAFs. The newly developed FAP-targeted bionic nanosystem, FAP-C NPs, induces the transition of activated CAFs to a quiescent state by delivering calcipotriol, thereby suppressing tumor stemness and lifting immune suppression. When combined with radiotherapy, this approach can double CD8^+^ T-cell infiltration and achieve a tumor suppression rate of 78.3% [105].

In addition to the rapid expansion of applications in PET imaging for cancer, FAP-targeted radionuclide therapy strategies are also attracting increasing attention. With the continuous optimization of FAPI ligand structures and the accumulation of data from multiple first-in-human studies and compassionate use programs, FAPI radioligand therapy has demonstrated good tolerability and preliminary efficacy in various advanced solid tumors. Table 1 summarizes key data from current representative FAPI radionuclide therapy studies.

### 4.4. Targeting CAF-Derived Extracellular Vesicles

Exosomes, as key carriers for communication between CAFs and tumor cells, have emerged in recent years as a novel target for overcoming radiation resistance. Unlike soluble factors, exosomes can simultaneously carry multiple bioactive molecules, enabling more efficient information transfer.

Studies have shown that targeting CAF-derived exosomes or the miR-590-3p they carry is a potential therapeutic strategy for overcoming radioresistance in colorectal cancer; this approach can inhibit DNA damage repair and promote tumor cell apoptosis, thereby restoring sensitivity to radiotherapy [83]. Furthermore, radiotherapy-induced CD44v3^+^ vesicles drive tumor stemness and treatment resistance by activating IL-6 signaling in fibroblasts; blocking vesicle generation via targeting the ESCRT pathway or disrupting their interaction with fibroblasts through heparan sulfate proteolysis can reverse this effect. Notably, CAF-EVs serve not only as therapeutic targets but also as biomarkers of radiotherapy response [85].

## 5. Future Perspectives

Based on an in-depth understanding of the biological functions of CAFs, targeting CAFs has become a key strategy for enhancing the efficacy of radiotherapy. Current research directions include targeting soluble factors, such as TGF-β, IL-1, IL-6, CXCL1, etc., blocking key molecules in the CAF-tumor cell dialogue, such as SFRP2, senescent-like CAFs, and FAK, utilizing FAP for precise delivery, including FAP-targeted immunotherapy, FAPα vaccines, and biomimetic nanosystems, as well as interfering with exosome-mediated intercellular communication. These strategies have demonstrated promising sensitizing effects in preclinical studies, and some have already entered the clinical trial phase, as summarized in Table 2.

Furthermore, the high heterogeneity of CAFs means that different subpopulations respond differently to treatment, offering new insights for precision interventions. With the continuous advancement of single-cell sequencing and spatial transcriptomics technologies, our understanding of CAF heterogeneity is gradually deepening. Multiple functionally distinct CAF subpopulations have been identified in various tumors, and their compositional patterns are closely associated with patient prognosis and treatment response. For example, in pancreatic cancer, periostin^+^ CAFs are associated with an invasive phenotype and poor prognosis, whereas podoplanin^+^ CAFs exhibit immune-related characteristics [121]; in ESCC, the MMP13^+^ and COL11A1^+^ myofibroblastic CAF subtypes are associated with poorly differentiated invasion and lymph node metastasis, respectively, and both predict poor prognosis [122]. Risk models based on CAF signature genes have been shown to independently predict patient survival and radiotherapy response in prostate cancer and lung adenocarcinoma; specifically, the prostate cancer model successfully generated survival curves predicting 2-, 3-, and 5-year survival rates [101,123]. A comparison of cancer types further revealed differences in the characteristics of CAFs between breast cancer and prostate cancer—the former are primarily enriched in the adhesion-mediated metastasis pathway, while the latter are associated with the immune response. These findings suggest that incorporating CAF molecular characteristics into patient stratification systems may provide a basis for personalized treatment strategies combining radiation therapy with CAF-targeted therapies.

In the longer term, strategies targeting CAFs need to move beyond the traditional approach of “targeted elimination.” Given the protective role of CAFs in maintaining normal tissue homeostasis, broad-spectrum elimination could lead to significant side effects. A more promising approach is to shift toward “functional reprogramming”—that is, using small-molecule drugs, epigenetic regulation, or nanodelivery systems to reprogram tumor-promoting CAFs into quiescent or tumor-suppressive phenotypes. Combining such reprogramming strategies with radiation therapy holds promise for achieving a therapeutic effect that turns the enemy into a friend.

At the same time, given that CAF mediates radioresistance through multiple synergistic mechanisms, a single-target strategy may struggle to fully reverse its tumor-promoting effects. Exploring the combination of CAF-targeted therapy with immune checkpoint inhibitors, chemotherapy, and anti-angiogenic therapy to determine the optimal timing, sequence, and dosage combinations represents an important direction for future research. Moving beyond the concept of targeting CAF alone, exploring combined strategies that simultaneously target CAF-immune checkpoint, CAF-angiogenesis, or CAF-matrix mechanical signaling pathways holds promise for achieving a global remodeling of the microenvironment.

In terms of biomarkers, circulating tumor cells, CAF-derived exosomes, and FAPI-PET/CT radiomic features can all serve as non-invasive predictors of radiotherapy efficacy. Integrating these multidimensional data into AI-driven predictive models holds promise for real-time assessment of radiotherapy response and personalized treatment adjustments.

Finally, most current strategies targeting CAFs to enhance radiosensitivity remain in the preclinical stage, and accelerating clinical translation is a top priority. Prospective candidate strategies should be prioritized for advancement into early-phase clinical trials, with predefined CAF biomarker stratification incorporated into trial designs. At the same time, it is essential to systematically evaluate the effects of long-term CAF intervention on normal tissue repair, wound healing, and immune function to ensure the safety of the therapeutic window.

## 6. Conclusions

In summary, CAFs are a key driver of TME remodeling following radiotherapy. Radiotherapy not only induces a fate transition in CAFs from survival to senescence but, more importantly, reshapes their function, enabling them to synergistically mediate radiation resistance through multiple mechanisms, including immunosuppression, paracrine pro-survival signaling, exosome-mediated communication, and matrix remodeling with bystander effects. Notably, the immunosuppressive function of CAFs persists even after high-dose radiotherapy, as irradiated CAFs continue to secrete inhibitory molecules and fail to trigger immunogenic cell death. These findings establish CAFs as a critical therapeutic barrier.

Consequently, targeting CAFs has emerged as a rational combination strategy to overcome radioresistance. While various approaches have shown promise in preclinical settings, the field is now shifting from proof of concept to the more nuanced challenge of precision intervention. The central question has evolved from “whether it is effective” to “how to achieve precision.” By leveraging CAF heterogeneity for patient stratification, advancing functional reprogramming over broad elimination, and accelerating clinical translation of the most promising candidates, CAF targeted therapy is poised to become an integral component of next generation radiotherapy combinations, ultimately delivering greater clinical benefits to cancer patients.

## Figures and Tables

**Table 1:** Research on FAPI-Targeted radionuclide therapy for tumors.

Strategy	Cancer Type	Population	Reference
^177^Lu-FAP-2286	Pancreatic cancer, breast cancer, colorectal cancer, ovarian cancer	11 patients	[106]
^90^Y-FAPI-46	Osteosarcoma, pancreatic cancer	9 patients	[107]
^177^Lu-FAP-2286	Lung cancer	9 patients	[108]
^177^Lu-DOTAGA.(SA.FAPi)_2_	Thyroid cancer	15 patients	[109]
^177^Lu-FAPI04	Breast cancer, thymic carcinoma, thyroid cancer, ovarian cancer	4 patients	[110]
^90^Y-FAPI-46	Solitary fibrous tumor (SFT)	11 patients	[111]
^90^Y-FAPI-46	metastatic SFT	3 patients	[112]
^213^Bi-FAPI-46	Colon cancer, anal cancer, breast cancer, prostate cancer	6 patients	[113]
[^177^Lu]Lu-DOTAGA.Glu.(FAPi)_2_	Thyroid cancer	1 patient	[114]
[^177^Lu]Lu-DOTAGA.FAPi	Breast cancer	19 patients	[115]
^177^Lu-EB-FAPI(^177^Lu-LNC1004)	Thyroid cancer	12 patients	[116]

**Table 2:** Clinical and preclinical studies on radiotherapy sensitization strategies targeting CAFs.

Target	Strategy with RT	Type	Reference
IL-1	Anakinra (IL-1 inhibitor)	Clinical trial	[97]
FAK	FAK Inhibition	Clinical trial	[71]
Farnesylated form of RhoB	Tipifarnib (farnesyltransferase inhibitor)	Clinical trial	[117]
PDGFRb	Predictive markers	Clinical trial	[118,119]
CAF/desmoplastic TME	Pulsed low-dose-rate radiation (PLDR)	Clinical trial	[120]
IL-6	Tocilizumab (anti-IL-6 monoclonal antibody)	Preclinical	[98]
Senescent-like CAFs	FOXO4-DRI (FOXO4-p53 interfering peptide)	Preclinical	[46]
FAP/CD40	FAP-CD40 antibody	Preclinical	[103]
FAPα	FAPα-based cancer vaccine	Preclinical	[104]
FAP	FAP-targeting biomimetic nanosystem	Preclinical	[105]

## Data Availability

No new data were created or analyzed in this study.

## Abbreviations

The following abbreviations are used in this manuscript:

TME	Tumor microenvironment
CAFs	Cancer-associated fibroblasts
α-SMA	α-smooth muscle actin
PDGFRα/β	Platelet-derived growth factor receptor α/β
FSP1	Fibroblast-specific protein 1
TGF-β	Transforming growth factor-beta
LIF	Leukemia Inhibitory Factor
BM-MSCs	Bone marrow-derived mesenchymal stem cells
EMT	Epithelial–mesenchymal transition
EndMT	Endothelial-mesenchymal transition
myCAFs	Myofibroblast-like CAFs
PDAC	Pancreatic ductal adenocarcinoma
HNSCC	Head and neck squamous cell carcinoma
iCAFs	Inflammatory CAFs
NSCLC	Non-small cell lung cancer
MET	Mesenchymal–epithelial transition
HGF	Hepatocyte growth factor
apCAFs	Antigen-presenting CAFs
mCAFs	Matrix CAFs
ilCAFs	Interferon-regulated CAFs
IRF1	Interferon regulatory factor 1
CAFR	Radioresistant cancer-associated fibroblasts
VEGF	Vascular endothelial growth factor
FGF	Fibroblast growth factor
SASP	Senescence-associated secretory phenotype
IL-1α	Interleukin-1 alpha
JAK/STAT	Janus kinase/signal transducer and activator of transcription
CXCL8	C-X-C motif chemokine ligand 8
SDF-1	Stromal Cell-Derived Factor-1
CXCR4	C-X-C chemokine receptor type 4
ROS	Reactive oxygen species
HIF-1α	Hypoxia-inducible factor-1α
MMP	Matrix metalloproteinase
PGE2	Prostaglandin E2
FAK	Focal adhesion kinase
IGF	Insulin-like growth factor
SOX4	SRY-box transcription factor 4
PDGF	Platelet-derived growth factor
EGF	Epidermal growth factor
Src	Sarcoma gene kinase
STAT3	Signal transducer and activator of transcription 3
NF-κB	Nuclear Factor kappa-B
ESCC	Esophageal squamous cell carcinoma
Col1	Collagen type 1
iNOS	Inducible nitric oxide synthase
NO	Nitric oxide
AKT	Threonine protein kinase
PTEN	Phosphatase and Tensin Homolog
PI3K	Phosphoinositide 3-kinase
CLCA4	Chloride channel accessory 4
TSP-2	Thrombospondin-2
bFGF	Basic fibroblast growth factor
SCC	Squamous cell carcinoma
MAPK	Mitogen-activated protein kinase
BA	Bintrafusp alpha
PD-L1	Programmed death-ligand 1
IL-1	Interleukin-1
SFRP2	Secreted Frizzled-Related Protein 2
PAI-1	Plasminogen Activator Inhibitor-1
FOXO4	Forkhead box protein O4
4-1BBL	4-1BB ligand
IFN-γ	Interferon-γ
CD40	Cluster of differentiation 40
cDC1	Conventional dendritic cell type 1
SABR	Stereotactic ablative radiotherapy
MDSCs	Myeloid-derived suppressor cells
Tregs	Regulatory T cells
ESCRT	Endosomal sorting complexes required for transport
